# Demographical, Clinical, and Psychological Characteristics of Users and Nonusers of an Online Platform for T2DM Patients (e-VitaDM-3/ZODIAC-44)

**DOI:** 10.1155/2016/6343927

**Published:** 2015-11-22

**Authors:** Yvonne Roelofsen, Michael van Vugt, Steven H. Hendriks, Kornelis J. J. van Hateren, Klaas H. Groenier, Frank J. Snoek, Nanne Kleefstra, Robbert Huijsman, Henk J. G. Bilo

**Affiliations:** ^1^Diabetes Centre, Isala, Zwolle, Netherlands; ^2^Department of Medical Psychology, VU University Medical Center and Academic Medical Center (AMC), Amsterdam, Netherlands; ^3^Department of General Practice, University of Groningen and University Medical Center Groningen, Groningen, Netherlands; ^4^Department of Internal Medicine, University of Groningen and University Medical Center Groningen, Groningen, Netherlands; ^5^Langerhans Medical Research Group, Zwolle, Netherlands; ^6^Institute of Healthcare Management & Policy, Erasmus University Rotterdam, Rotterdam, Netherlands

## Abstract

*Background*. Online platforms offer opportunities for support in changing lifestyle and taking responsibility for one's health, but engaging patients with type 2 diabetes is challenging. Previous studies have shown that patients interested in platforms were more often male, younger, and higher educated. This study aims to investigate differences in clinical and psychological characteristics between users and nonusers of a newly developed platform. *Methods*. A prospective study started in the Drenthe region of Netherlands. Participants in the study concerning quality of care and quality of life were additionally invited to use the platform. *Results*. 633 patients were registered after they opted for platform use. Of these patients, 361 (57.0%) never logged on, 184 (29.1%) were labeled “curious” users, and 88 (13.9%) were identified as “active” users. Users had lower HbA1c levels and more often hypertension compared to nonusers, and reported higher quality of life, better well-being, lower diabetes-related distress, and better medication adherence. *Discussion*. Platform use was associated with more favorable clinical and psychological characteristics relative to nonuse. Those with greater severity of disease, lower mood, and progression of disease used the platform the least. Other approaches need to be developed to reach these patients. Furthermore, improving the platform could also help to reach them. This trial is registered with Clinicaltrials.gov NCT01570140.

## 1. Background

Type 2 diabetes mellitus (T2DM) in itself is associated with poorer health-related quality of life (HRQoL) [1]. People with T2DM are susceptible to develop long term complications, such as retinopathy, neuropathy, nephropathy, and chronic heart disease, which negatively influence HRQoL [2]. To prevent or delay development of these long term complications, adequate treatment modalities are necessary which mainly involve lifestyle changes and pharmacological treatment. Adherence to medication prescription and implementing life style changes are often better maintained and facilitated, when patients consider themselves more responsible for their treatment and have more knowledge regarding the causes and consequences of their disease. Improvements in knowledge about their disease can be described as promotion of health literacy. e-Health applications, such as web-portals, teleconsultation, and online care platforms, have the potential to support patients in changing lifestyle and taking more responsibility for their own health [3]. However, varying effects on clinical outcomes, quality of life, degree of self-care, perceived stress levels, patient satisfaction, and costs have been reported [4–10].

Previous studies showed that patients who were interested in using an online care platform were more often male, younger, and higher educated [11, 12]. However, within the subgroup of interested patients these differences were not found between actual users and nonusers [11]. In addition, other factors associated with higher portal enrollment and utilization are higher income, nonblack race, higher self-efficacy, and having better regulated diabetes [13]. Identifying the differences between platform users and nonusers could provide information to help target and support nonusers in becoming more active in their diabetes self-management.

The aim of the present, explorative study was to investigate possible differences in demographic, clinical, and psychological characteristics between users and nonusers of the platform e-Vita.

## 2. Methods

### 2.1. Study Design

We performed a cross-sectional analysis of baseline data of users and nonusers of the online patient platform e-Vita. Data was obtained from a prospective observational cohort study. Detailed information about the methods and design of the study as a whole can be found elsewhere [14].

### 2.2. Study Population and Setting

Forty-three out of 110 general practices in the Drenthe region of the Netherlands invited their T2DM patients for participation in a prospective observational cohort study concerning quality of care and HRQoL. Patients were also invited to use the online care platform e-Vita, in addition to their usual treatment. Patients interested in using the platform were registered by their practice nurse (PN) and received a user ID. In this ongoing study, participants were recruited from May 2012 onward. The current analysis includes patients recruited from May 2012 till March 2014.

### 2.3. Measurements

Demographic and clinical data were obtained from the personal health record systems of the general practitioners (GP), based on a core dataset of T2DM related information as advised by the Dutch Diabetes Federation and the Dutch College of General Practitioners [14]. All T2DM patients participating in the study filled in a range of validated questionnaires concerning perceived quality of life measured by the EuroQol Five Dimension (EQ-5D) Scale [15–17], emotional well-being measured by the World Health Organization Wellbeing Index 5-Item (WHO-5) questionnaire [18, 19], diabetes-related distress measured by the Problem Areas in Diabetes 5-Item (PAID-5) questionnaire [20], diabetes self-care behavior measured by 7 Dimensions of the Summary of Diabetes Self-Care Activities (SDSCA) questionnaire [21], and quality of received care measured by the Europep [22]. Suboptimal emotional well-being was defined by a raw score lower than 13 on the WHO-5 [23]. Additional questions about smoking habits, employment, and educational background were also included. To identify users and nonusers, registration data from the application software and log-files were used.

### 2.4. Description of e-Vita Platform

The e-Vita platform for T2DM patients (accessible through the login button on https://www.e-vita.nl/) [11, 14, 24] contains the following components: (1) an overview of health data concerning annual check-ups from 2009 onward, (2) educational modules meant to support care through self-management by setting person-specific goals and actions [25], (3) prompting patient self-monitoring of clinical values, (4) educational modules aimed at increasing diabetes knowledge, and (5) providing reliable information on T2DM in general.

### 2.5. Users and Nonusers

Information about login status and log-data were used to group patients into nonusers and users. All patients who logged in at least once were considered as users. Patients who had been online for at least two sessions with a minimum of five minutes per session were defined as “active” users; other patients were defined as “curious” users. A session included all logins to the platform within thirty minutes [24].

### 2.6. Statistical Analyses

Statistical analyses were performed using SPSS version 20 (IBM Corporation, Somers, NY, USA). Quantitative variables are described in means and standard deviations when normally distributed; otherwise medians and interquartile ranges are also described. Categorical variables are described in numbers and percentages. To identify differences in the domains of interest between the different groups of users, the Linear Mixed Models procedure was used, with groups of users being fixed factors (nonusers being the reference group), while adjusting for age and sex. Fisher's exact test was used for categorical data. Differences were considered to be significant at a *p* value of <0.05. In addition, results are adjusted for age and gender. Because of the explorative design of this study, no corrections for multiple testing were made [26]. Instead, the calculated *p* values are only used as an indication of to what extent a difference could be interesting for further research.

### 2.7. Ethics

This study was approved by the Medical Ethical Review Committee of Isala, Zwolle, the Netherlands, and registered in Clinicaltrials.gov under number NCT01570140.

## 3. Results

**Figure 1:**
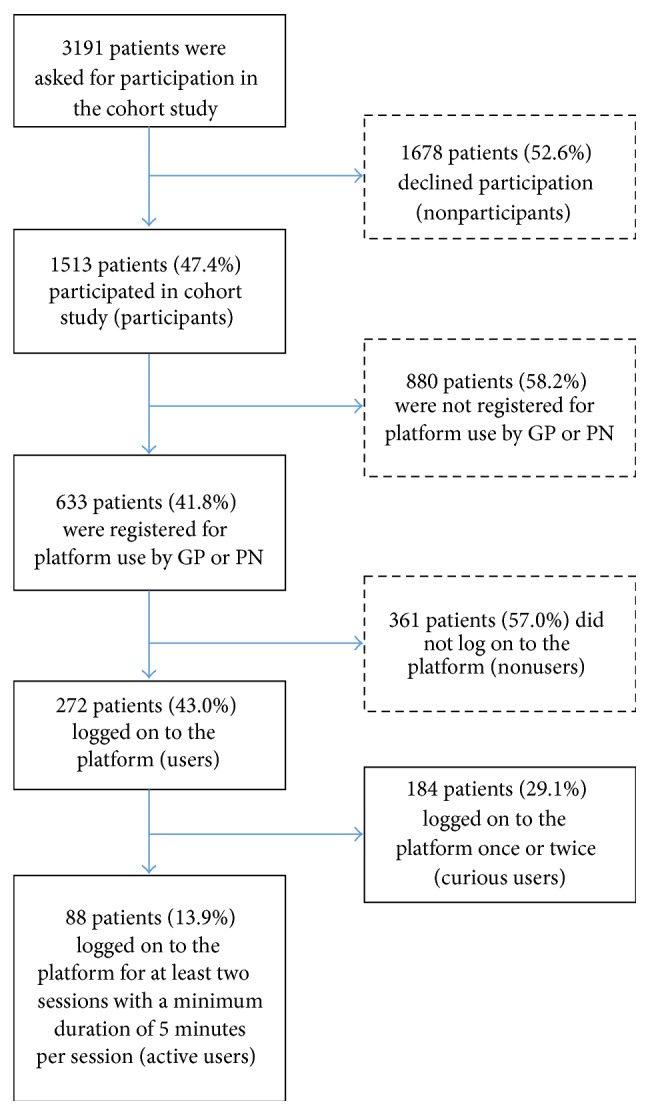
Flowchart of patients and definitions.

In the period from May 2012 to March 2014, 3191 patients were invited to participate in the cohort study and to use the e-Vita platform. 633 patients were registered for care platform use. See Figure 1 for the patient flow.

Table 1 shows all differences and other notable characteristics for the comparison between nonusers, curious users, and active users of the platform. No differences were found in demographical characteristics between nonusers, curious users, and active users. HbA1c level of nonusers was higher compared to curious users (*p* = 0.038) and to active users (*p* = 0.001). Curious and active users were more often known with hypertension compared to nonusers (*p* = 0.025). Curious users assessed the GP better on one question of the Europep compared to nonusers and active users (*p* = 0.047). Curious users scored higher on EQ-5D (*p* = 0.030) and EQ-VAS (0.032) compared to nonusers, with no significant differences between curious users and active users or nonusers and active users. In addition, curious users' WHO-5 score as well as their answers to the individual WHO-5 questions reported less depressive symptoms compared to nonusers and active users. Curious users scored lower on PAID-5 compared to nonusers (*p* = 0.016), with no significant differences between curious users versus active users and nonusers versus active users. Curious users performed better on one dimension of self-reported self-management activities (medication intake) compared to nonusers (*p* = 0.020), with no significant difference between curious users versus active users and nonusers versus active users.  Table 2 shows the Cronbach's alpha for all the multi-item scales.

See Appendix A for tables with all characteristics as mentioned in the methods section for the comparison between nonusers and users. See Appendix B for tables with all characteristics for the comparison between nonusers, curious users, and active users.

The differences in characteristics between nonusers, curious users, and active users have also been adjusted for age and gender in a multivariate analysis. The results are shown in Table 3. *p* values below 0.05 were found for differences regarding HbA1c between active users and nonusers (−3.624 mmol/mol) as well as between curious users and nonusers (−1.989 mmol/mol) and for differences between curious users and nonusers regarding EQ-5D (0.044), EQ-VAS (4.611), WHO-5 (3.609), PAID-5 (−0.929), and medication intake (0.236).

## 4. Discussion

In this exploratory study we found that only a small amount of clinical and psychological characteristics were associated with platform use. Curious users as well as active users had lower HbA1c compared to nonusers, which is in agreement with other studies [27, 28]. The more frequent presence of hypertension in curious and active users, however, contradicts with these studies. Curious users scored higher on EQ-5D and EQ-VAS and lower on PAID-5. Curious users scored also better on medication intake, which may reflect higher self-efficacy, in agreement with the study by Sarkar et al. [29]. After adjustment for age and gender, the difference in WHO-5 score between curious users and nonusers was also significant.

We observed that most of the patients, who were registered for platform use, never logged on. This could be influenced by (an insufficient) intrinsic motivation and (no) intention to change behaviours. Another explanation could be that patients do not see the platform as useful or as an added value to regular treatment. As an alternative explanation, login procedures might be too difficult and after trying for some time they might give up.

Previous research showed that web-portals and online care platforms are susceptible to implementation problems, low participation rates, and nonadherence, which, amongst others, can be caused by a mismatch in expectations between software developers, health care providers, and users [30–37]. Other reasons for limited use of care platforms or nonadherence rates are as follows: abundance of functionalities on a platform, no connection with the needs of patients, implementation by management only without active involvement of care providers, no embedding in the regular care process, no space for habituation, underestimation of the complexity of lifestyle changes in general [38], and barriers to easy access to a portal (e.g., complicated login procedures). Despite the use of focus groups for designing and testing, these reasons might also be applicable to the e-Vita platform and improvements could be made.

The current study has some limitations. A preselection of participants could in part have influenced results. Only patients who expressed their interest received a user-ID [14]; see also Figure 1. Relevant and significant differences might be more difficult to find.

Data were not complete for all patients, especially with regard to complications and risk factors (complete for 50–60%; see Tables 6 and 11). This may have led to an underestimation of presence of complications and risk factors. In addition, not all patients were seen by their GP or PN for the regular yearly check-up in the year 2012, which contributed to missing values in clinical parameters. Some questions about the assessment of the general practice and the general practitioner were poorly answered in general. A reason for this could be social desirability; patients may not like to be negative about their GP and prefer not answering these questions.

Although the online care platform e-Vita was designed for being suitable for all T2DM patients, a general assumption is that those with greater severity of disease, lower mood, progression of the disease, and complications would probably benefit most from an online care platform. However, when assessing the presented results, these patients use the platform the least.

Possibly, the current users were already more in control of their life and health and could therefore be more open to other forms of support, including e-Health facilities. Challenges to reach other patients remain manifold. A patients' passive attitude may not be overcome by only providing e-facilities, since one's interest and the sense of disease burden are low or even absent in the majority of the T2DM population. Factors as knowledge, motivation, and intention could be considered in future research.

## Figures and Tables

**Table 1:** Differences and notable characteristics of nonusers, curious users, and active users.

*n* (%) or mean (SD) Demographic and clinical parameters	Nonusers (*n* = 361)	Missing	Curious users (*n* = 184)	Missing	Active users (*n* = 88)	Missing	Univariate *p* value
Men	214 (59.3)	0 (0)	113 (61.4)	0 (0)	50 (56.8)	0 (0)	0.760
Age in years	62.1 ± 9.5 63.0 (56.5–68.0)	0 (0)	61.8 ± 9.5 62.0 (56.3–68.0)	0 (0)	62.0 ± 9.4 63.0 (57.0–67.0)	0 (0)	0.935
Ethnicity							
Caucasian	292 (99.0)	66 (18.3)	143 (100)	41 (22.3)	65 (100)	23 (6.1)	0.706
Other	3 (1.0)		0 (0)		0 (0)		0.382
Employment							
Fulltime/part-time working	99 (34.3)	72 (19.9)	61 (39.6)	30 (16.3)	20 (23.8)	4 (4.5)	0.063
Retired	134 (46.4)		70 (45.5)		46 (54.8)		
Unemployed/housekeeper	38 (13.1)		20 (13.0)		9 (10.7)		
Incapacitated	18 (6.2)		3 (1.9)		9 (10.7)		
Educational level							
None	0 (0)	73 (20.2)	1 (0.7)	31 (16.8)	0 (0)	4 (4.5)	0.125
Primary school	24 (8.3)		9 (5.9)		4 (4.8)		
Low	127 (44.1)		52 (34.0)		27 (32.1)		
Intermediate	86 (29.9)		51 (33.3)		30 (35.7)		
High	51 (17.7)		40 (26.1)		23 (27.4)		
T2DM duration in years	6.2 ± 4.6 6.0 (2.0–9.0)	9 (2.5)	5.7 ± 4.4 5.0 (2.0–8.0)	1 (0.5)	5.4 ± 4.4 4.5 (2.0–8.0)	0 (0)	0.165
HbA1c in mmol/mol	50.6 ± 9.5 50.0 (45.0–54.0)	3 (0.8)	48.7 ± 7.4 48.0 (43.0–54.0)	0 (0)	47.0 ± 7.0 46.0 (43.0–50.8)	0 (0)	**0.001**
BMI	29.8 ± 4.9 29.0 (26.5–32.5)	3 (0.8)	30.0 ± 4.8 29.3 (26.9–32.3)	0 (0)	29.9 ± 8.0 28.0 (26.0–32.6)	2 (2.3)	0.921

Comorbidities/complications							
Hypertension	191 (84.1)	134 (37.1)	113 (93.4)	63 (34.2)	51 (92.7)	33 (37.5)	**0.025**

Items of Europep: patients who scored 4 (good) to 5 (excellent) What is your assessment of the general practitioner over the last 12 months with respect to the following?							
Making it easy for you to tell him or her about your problem	345 (93.5)	18 (4.7)	187 (97.9)	17 (8.2)	84 (93.3)	2 (2.2)	**0.047**

*EQ-5D* index-score	0.9 ± 0.2	72 (19.9)	0.9 ± 0.1	32 (17.4)	0.9 ± 0.2	4 (4.5)	**0.030**
*EQ-VAS *	74.7 ± 17.4 80.0 (60.0–90.0)	74 (20.5)	79.3 ± 13.8 80.0 (73.0–90.0)	35 (19.0)	76.9 ± 16.5 80.0 (0.0–90.0)	5 (5.7)	**0.032**

*WHO-5* score indicates suboptimal well-being, screening depression advised	36 (12.6)	76 (21.1)	8 (5.3)	33 (17.9)	9 (10.8)	1 (1.1)	**0.018**
*WHO-5* answers advise screening depression	43 (15.5)	76 (21.1)	6 (4.0)	33 (17.9)	11 (13.3)	1 (1.1)	**0.002**

*PAID-5* total score	2.8 ± 3.1 2.0 (0.0–4.5)	76 (21.1)	1.8 ± 2.4 1.0 (0.0–3.0)	32 (17.4)	2.2 ± 2.5 1.0 (0.0–4.0)	1 (1.1)	**0.016**

*SDSCA *							
Medication in number of days	6.7 ± 1.0 7.0 (7.0–7.0)	73 (20.2)	7.0 ± 0.2 7.0 (7.0–7.0)	30 (16.3)	6.8 ± 0.8 7.0 (7.0–7.0)	4 (4.5)	**0.020**

**Table 2:** Cronbach's alpha for multi-item scales.

Multi-item scale	*α*
Europep	
Total	0.963
Subscale general practice	0.966
Subscale general practitioner	0.840
EQ-5D	0.652
WHO-5	0.872
PAID-5	0.867
SDSCA	
Total	0.517
Subscale general diet	0.875
Subscale specific diet	0^1^
Subscale total diet	0.446^2^
Subscale exercise	0.663
Subscale blood-glucose testing	0.912
Subscale foot-care	0.593

^1^Because of the negative intercorrelation between the two items Cronbach's alpha is reported to be 0.

^2^The alpha for the subscale total diet is lower than that for the subscale general diet due to the low reliability of the subscale specific diet.

**Table 3:** Results of multivariate analysis, adjusted for age and gender.

	*b*-coefficient	95% CI		*p* value
		Lower bound	Upper bound	
T2DM duration in years				
Intercept	0.018	−2.325	2.360	0.988
Platform use				0.186
Active users	−0.845	−1.876	0.186	0.108
Curious users	−0.511	−1.300	0.277	0.203
Nonusers	Ref. Cat.			
Male	−0.121	−0.832	0.589	0.738
Female	Ref. Cat.			
Age	0.101	0.064	0.138	<0.0005
HbA1c in mmol/mol				
Intercept	53.431	48.931	57.931	<0.0005
Platform use				<0.0005
Active users	−3.624	−5.627	−1.621	**<0.0005**
Curious users	−1.989	−3.516	−0.462	**0.011**
Nonusers	Ref. Cat.			
Male	1.103	−0.270	2.477	0.115
Female	Ref. Cat.			
Age	−0.055	−0.127	0.016	0.126
BMI				
Intercept	37.430	34.658	40.202	<0.0005
Platform use				0.924
Active users	0.079	−1.159	1.317	0.900
Curious users	0.189	−0.747	1.124	0.692
Nonusers	Ref. Cat.			
Male	−1.087	−1.931	−0.244	0.012
Female	Ref. Cat.			
Age	−0.113	−0.156	−0.069	<0.0005
EQ-5D				
Intercept	0.866	0.773	0.958	<0.0005
Platform use				0.022
Active users	0.008	−0.031	0.047	0.674
Curious users	0.044	0.013	0.076	**0.006**
Nonusers	Ref. Cat.			
Male	0.056	0.027	0.085	<0.0005
Female	Ref. Cat.			
Age	−0.001	−0.002	0.001	0.343
EQ-VAS				
Intercept	71.007	61.663	80.350	<0.0005
Platform use				0.019
Active users	2.291	−1.691	6.275	0.259
Curious users	4.611	1.384	7.838	**0.005**
Nonusers	Ref. Cat.			
Male	2.977	0.095	5.859	0.043
Female	Ref. Cat.			
Age	0.030	−0.118	0.178	0.690
WHO-5				
Intercept	58.138	48.911	67.365	<0.0005
Platform use				0.065
Active users	−0.089	−4.008	3.829	0.964
Curious users	3.609	0.446	6.773	**0.025**
Nonusers	Ref. Cat.			
Male	5.766	2.932	8.600	<0.0005
Female	Ref. Cat.			
Age	0.142	−0.004	0.289	0.057
PAID-5				
Intercept	5.129	3.520	6.737	<0.0005
Platform use				0.004
Active users	−0.511	−1.195	0.173	0.143
Curious users	−0.929	−1.480	−0.378	**0.001**
Nonusers	Ref. Cat.			
Male	−0.143	−0.639	0.353	0.571
Female	Ref. Cat.			
Age	−0.037	−0.062	−0.011	0.005
SDSCA-medication				
Intercept	6.087	5.575	6.600	<0.0005
Platform use				0.028
Active users	0.081	−0.132	0.296	0.458
Curious users	0.236	0.063	0.408	**0.008**
Nonusers	Ref. Cat.			
Male	0.096	−0.058	0.250	0.222
Female	Ref. Cat.			
Age	0.010	0.002	0.018	0.020

**Table 4:** Demographic and clinical characteristics of users and nonusers.

Demographic and clinical parameters *n* (%)/mean ± SD/median (25–75 quartiles)	Nonusers (*n* = 361)	Missing	Users (*n* = 272)	Missing	Univariate *p* value
Men	214 (59.3)	0 (0)	163 (59.9)	0 (0)	0.95
Age in years	62.1 ± 9.5 63.0 (56.5–68.0)	0 (0)	61.8 ± 9.4 62.5 (57.0–68.0)	0 (0)	0.732
Ethnicity					
Caucasian	292 (99.0)	66 (18.3)	208 (100)	64 (2.5)	0.271
Other	3 (1.0)		0 (0)		
T2DM duration in years	6.2 ± 4.6 6.0 (2.0–9.0)	9 (2.5)	5.6 ± 4.4 5.0 (2.0–8.0)	1 (0.4)	0.068
HbA1c in mmol/mol	50.6 ± 9.5 50.0 (45.0–54.0)	3 (0.8)	48.2 ± 7.3 47.0 (43.0–53.0)	0 (0)	**<0.0005**
BMI	29.8 ± 4.9 29.0 (26.5–32.5)	3 (0.8)	30.0 ± 6.0 28.7 (26.3–32.4)	2 (0.7)	0.724
Systolic blood pressure in mmHG	135.6 ± 15.5	0 (0)	136.5 ± 16.0	4 (1.5)	0.463
Cholesterol in mmol/L	4.4 ± 1.0	4 (1.1)	4.4 ± 0.9	2 (0.7)	0.499
HDL in mmol/L	1.3 ± 0.4	4 (1.1)	1.3 ± 0.4	3 (1.1)	0.581
Cholesterol/HDL ratio	3.6 ± 1.1	160 (44.3)	3.6 ± 1.3	92 (33.8)	0.899
LDL in mmol/L	2.4 ± 0.9	12 (3.3)	2.3 ± 0.8	6 (2.2)	0.240
Triglycerides in mmol/L	1.7 ± 1.0 1.5 (1.0–2.1)	7 (1.9)	1.8 ± 1.2 1.5 (1.1–2.1)	2 (0.7)	0.482
Creatinine in *µ*mol/L	78.6 ± 17.2 77.0 (67.0–88.0)	6 (1.7)	79.9 ± 17.5 79.0 (67.0–90.0)	1 (0.4)	0.359
Alb./creat. ratio in mg/mmol					
Men	2.0 ± 4.4 0.7 (0.3–1.5)	23 (10.7)	1.9 ± 5.8 0.5 (0.3–1.5)	25 (15.3)	0.853
Women	1.6 ± 3.5 0.7 (0.3–1.5)	31 (21.1)	0.9 ± 1.1 0.6 (0.4–1.2)	18 (16.5)	0.070
MDRD in mL/min/1.73 m^2^	79.1 ± 49.0 75.0 (61.0–88.0)	5 (1.4)	76.0 ± 16.6 74.0 (61.0–87.0)	1 (0.4)	0.329
Smoking					
Yes	54 (15.1)	3 (0.8)	41 (15.1)	1 (0.4)	0.306
Before	158 (44.1)		104 (38.4)		
No	146 (40.8)		126 (46.5)		
Alcohol consumption in units/day					
0	166 (58.9)	79 (21.9)	139 (60.7)	43 (15.8)	0.870
1	61 (21.6)		52 (22.7)		
2	39 (13.8)		30 (13.1)		
3	11 (3.9)		7 (3.1)		
4	4 (1.4)		1 (0.4)		
5	0 (0)		0 (0)		
6	1 (0.4)		0 (0)		
Employment					
Fulltime/part-time working	99 (34.3)	72 (19.9)	81 (34.0)	34 (12.5)	0.909
Retired	134 (46.4)		116 (48.7)		
Unemployed/ housekeeper	38 (13.1)		29 (12.2)		
Incapacitated	18 (6.2)		12 (5.0)		
Educational level					
None	0 (0)	73 (20.2)	1 (0.4)	35 (12.9)	**0.017**
Primary school	24 (8.3)		13 (5.5)		
Low	127 (44.1)		79 (33.3)		
Intermediate	86 (29.9)		81 (29.8)		
High	51 (17.7)		63 (23.2)		

**Table 5:** Medication use of users and nonusers.

Medication prescription *n* (%)	Nonusers (*n* = 361)	Missing	Users (*n* = 272)	Missing	Univariate *p* value
Diabetes-related					
Oral treatment only	251 (71.3)	9 (2.5)	192 (71.9)	5 (1.8)	0.702
Insulin treatment only	4 (1.1)	9 (2.5)	1 (0.4)	5 (1.8)	0.931
Mix of oral and insulin treatment	40 (11.4)	9 (2.5)	23 (8.6)	5 (1.8)	0.248
No medication	57 (16.2)	9 (2.5)	51 (19.1)	5 (1.8)	0.417
Comorbidity or complication related					
Calcium channel blockers	50 (14.2)	9 (2.5)	47 (17.6)	5 (1.8)	0.236
Beta blockers	128 (36.4)	9 (2.5)	110 (41.2)	5 (1.8)	0.145
Diuretics	121 (34.4)	9 (2.5)	94 (35.2)	5 (1.8)	0.870
Ace and RAAS inhibitors	196 (55.7)	9 (2.5)	141 (52.8)	5 (1.8)	0.480
Other blood pressure lowering medications	3 (0.9)	9 (2.5)	1 (0.4)	5 (1.8)	0.637
Lipid lowering medication	280 (79.5)	9 (2.5)	213 (79.8)	5 (1.8)	0.847

**Table 6:** Complications and risk factors of users and nonusers.

Complications and risk factors *n* (%)	Nonusers (*n* = 361)	Missing	Users (*n* = 272)	Missing	Univariate *p* value
Cardiovascular, total	225 (96.2)	127 (35.2)	187 (98.4)	82 (30.1)	0.240
Cardiovascular, specific					
Angina pectoris	41 (21.7)	172 (47.6)	28 (19.7)	130 (47.8)	0.787
Myocardial infarct	29 (15.3)	172 (47.6)	23 (16.0)	128 (47.1)	0.880
Other/chronic ischemic heart diseases	34 (16.1)	150 (41.6)	24 (13.6)	96 (35.3)	0.569
Hypertension	191 (84.1)	134 (37.1)	164 (93.2)	96 (35.3)	**0.008**
TIA	12 (6.4)	174 (48.2)	7 (5.0)	133 (48.9)	0.642
CVA	13 (7.0)	176 (48.8)	10 (7.1)	132 (48.5)	1.000
Intermittent claudication	7 (3.3)	150 (41.6)	7 (4.0)	96 (35.3)	0.788
Aortic aneurysms	4 (1.9)	150 (41.6)	2 (1.1)	96 (35.3)	0.693
CABG	15 (5.1)	68 (18.8)	11 (5.4)	68 (25.0)	1.000
PTCA	28 (9.6)	68 (18.8)	14 (6.8)	67 (24.6)	0.327
Heart failure	14 (8.1)	189 (52.4)	10 (7.4)	136 (50.0)	0.834
Retinopathy	19 (9.3)	156 (43.2)	18 (10.2)	95 (34.9)	0.863
Renal impairment	35 (18.6)	173 (47.9)	26 (18.6)	132 (48.5)	1.000
Albuminuria					
Men	30 (14.5)	7 (3.3)	20 (12.6)	4 (2.5)	0.647
Women	8 (5.8)	8 (5.4)	1 (1.0)	5 (4.6)	0.082
Neuropathy	49 (22.2)	140 (38.8)	39 (22.4)	98 (36.0)	1.000
Foot complication					
SIMMs 0	228 (77.6)	67 (18.6)	161 (76.7)	62 (22.8)	0.783
SIMMs 1	57 (19.4)		40 (19.0)		
SIMMs 2 or 3	9 (3.1)		9 (4.3)		
Psychiatric disorders	19 (9.0)	150 (41.6)	9 (5.1)	96 (35.3)	0.124

SIMMS refers to risk factors in the diabetic foot, the number is the stage which ranges from 0–3.

0: no loss of protective sensibility (PS) & Peripheral arterial disease (PAV).

1: loss of PS or PAV, with no signs of increased local pressure.

2: loss of PS in combination with and/or PAV and/or signs of local elevated pressure.

3: ulcer or amputation in history.

**Table 7:** Scores on quality of care (Europep) of users and nonusers.

Items of Europep: patients who scored 4 (good) or 5 (excellent) *n* (%)	Nonusers (*n* = 361)	Missing	Users (*n* = 272)	Missing	Univariate *p* value
*What is your assessment of the general practitioner over the last 12 months with respect to the following? *					
Making you feel you have time during consultation	337 (97.4)	15 (4.2)	256 (98.1)	11 (4.0)	0.622
Showing interest in your personal situation	324 (94.5)	18 (5.0)	246 (94.6)	12 (4.4)	0.864
Making it easy for you to tell him or her about your problem	323 (93.4)	15 (4.2)	245 (96.5)	18 (6.6)	0.110
Involving you in decisions about your medical care	311 (92.3)	24 (6.6)	239 (94.1)	18 (6.6)	0.290
Listening to you	322 (92.3)	12 (3.3)	243 (94.6)	15 (5.5)	0.270
Keeping your records and data confidential	310 (95.7)	37 (10.2)	236 (95.9)	26 (9.6)	0.846
Providing quick relief of your symptoms	272 (87.5)	50 (13.9)	201 (85.9)	38 (14.0)	0.635
Helping you to feel well so that you can perform your normal daily activities	265 (89.8)	66 (18.3)	196 (91.2)	57 (21.0)	0.483
Thoroughness of the approach to your problems	308 (91.4)	24 (6.6)	227 (89.7)	19 (7.0)	0.786
Your physical examination	292 (90.1)	37 (10.2)	222 (92.1)	31 (11.4)	0.327
Offering services for preventing diseases (screening, health checks, and immunizations)	286 (91.4)	48 (13.3)	225 (92.6)	29 (10.7)	0.655
Explaining the purpose of examinations, tests, and treatments	307 (93.0)	31 (8.6)	240 (93.8)	16 (5.9)	0.518
Telling you enough about your symptoms and/or illness	306 (92.2)	29 (8.0)	238 (93.3)	17 (6.3)	0.448
Helping you deal with emotions related to your health status	198 (86.8)	133 (36.8)	133 (84.7)	115 (42.3)	0.888
Helping understand why it is important to follow the GP's advice	295 (89.7)	32 (8.9)	219 (89.4)	27 (9.9)	0.894
Knowing what has been done or told during previous contacts in the practice	270 (84.9)	43 (11.9)	219 (89.4)	27 (9.9)	0.071
Preparing you for what to expect from specialists, hospital care, and other care providers	199 (85.4)	128 (35.5)	156 (83.5)	85 (31.3)	0.513

*What is your assessment of the general practice over the last 12 months with respect to the following? *					
The helpfulness of the practice staff (other than the doctor) to you	313 (93.4)	26 (7.2)	235 (92.9)	19 (7.0)	0.878
Getting an appointment to suit you	301 (88.5)	21 (5.8)	224 (86.5)	13 (4.8)	0.639
Getting through to the practice on telephone	249 (73.0)	20 (5.5)	180 (69.5)	13 (4.8)	0.662
Being able to talk to the general practitioner on the telephone	167 (70.5)	124 (34.3)	106 (63.1)	104 (38.2)	0.150
Waiting time in the waiting room	246 (71.3)	16 (4.4)	170 (65.4)	12 (4.4)	0.175
Providing quick services for urgent health problems	241 (90.3)	94 (26.0)	171 (86.8)	75 (27.6)	0.398

**Table 8:** Scores on quality of life (EQ-5D), well-being (WHO-5), diabetes-related distress (PAID-5), and self-care behavior (SDSCA).

EQ-5D, WHO-5, PAID-5, and SDSCA *n* (%)/mean ± SD/median (25–75 quartiles)	Nonusers (*n* = 361)	Missing	Users (*n* = 272)	Missing	Univariate *p* value
EQ-5D index-score	0.9 ± 0.2	72 (19.9)	0.9 ± 0.1	36 (13.2)	**0.028**
EQ-VAS	74.7 ± 17.4 80.0 (60.0–90.0)	74 (20.5)	78.4 ± 14.9 80.0 (71.0–90.0)	40 (14.7)	**0.014**
WHO-5 index-score	70.4 ± 17.9 76.0 (60.0–80.0)	76 (21.1)	72.7 ± 14.2 76.0 (68.0–80.0)	38 (14.0)	0.096
WHO-5 score indicates suboptimal well-being, screening depression advised	36 (12.6)	76 (21.1)	17 (7.3)	38 (14.0)	**0.018**
WHO-5 answers advise screening depression	43 (15.5)	76 (21.1)	17 (7.3)	38 (14.0)	**0.004**
PAID-5 total score	2.8 ± 3.1 2.0 (0.0–4.5)	76 (21.1)	2.0 ± 2.5 1.0 (0.0–3.0)	38 (14.0)	**0.005**
PAID-5 score indicates distress	15 (5.3)	76 (21.1)	6 (2.6)	38 (14.0)	0.058
SDSCA					
General diet in number of days	5.4 ± 1.8 6.0 (5.0–7.0)	76 (21.1)	5.6 ± 1.8 6.0 (5.0–7.0)	37 (13.6)	0.269
Specific diet in number of days	5.6 ± 1.1 5.7 (4.7–6.3)	73 (20.2)	5.7 ± 1.0 6.0 (5.3–6.7)	34 (12.5)	0.056
Exercise in number of days	4.0 ± 2.0 4.0 (2.5–5.5)	72 (19.9)	4.0 ± 1.8 4.0 (2.5–5.5)	34 (12.5)	0.919
Blood-glucose in number of days	2.1 ± 2.2 1.0 (0.0–4.0)	74 (20.5)	2.0 ± 2.2 1.0 (0.5–3.5)	34 (12.5)	0.675
Foot-care in number of days	1.9 ± 2.0 1.5 (0.0–3.5)	72 (19.9)	1.9 ± 2.0 1.0 (0.0–3.5)	34 (12.5)	0.695
Medication in number of days	6.7 ± 1.0 7.0 (7.0–7.0)	73 (20.2)	6.9 ± 0.5 7.0 (7.0–7.0)	34 (12.5)	**0.013**
Smoking	54 (25.1)	146 (40.4)	38 (22.8)	105 (38.6)	**0.418**

**Table 9:** Demographic and clinical characteristics of curious users, active users, and nonusers.

Demographic and clinical parameters *n* (%)/mean ± SD/median (25–75 quartiles)	Nonusers (*n* = 361)	Missing	Curious users (*n* = 184)	Missing	Active users (*n* = 88)	Missing	Univariate *p* value
Men	214 (59.3)	0 (0)	113 (61.4)	0 (0)	50 (56.8)	0 (0)	0.760
Age in years	62.1 ± 9.5 63.0 (56.5–68.0)	0 (0)	61.8 ± 9.5 62.0 (56.3–68.0)	0 (0)	62.0 ± 9.4 63.0 (57.0–67.0)	0 (0)	0.935
Ethnicity							
Caucasian	292 (99.0)	66 (18.3)	143 (100)	41 (22.3)	65 (100)	23 (6.1)	0.706
Other	3 (1.0)		0 (0)		0 (0)		0.382
T2DM duration in years	6.2 ± 4.6 6.0 (2.0–9.0)	9 (2.5)	5.7 ± 4.4 5.0 (2.0–8.0)	1 (0.5)	5.4 ± 4.4 4.5 (2.0–8.0)	0 (0)	0.165
HbA1c in mmol/mol	50.6 ± 9.5 50.0 (45.0–54.0)	3 (0.8)	48.7 ± 7.4 48.0 (43.0–54.0)	0 (0)	47.0 ± 7.0 46.0 (43.0–50.8)	0 (0)	**0.001**
BMI	29.8 ± 4.9 29.0 (26.5–32.5)	3 (0.8)	30.0 ± 4.8 29.3 (26.9–32.3)	0 (0)	29.9 ± 8.0 28.0 (26.0–32.6)	2 (2.3)	0.921
Systolic blood pressure in mmHG	135.6 ± 15.5	0 (0)	137.2 ± 16.3	2 (1.1)	135.1 ± 15.3	2 (2.3)	0.463
Cholesterol in mmol/L	4.4 ± 1.0	4 (1.1)	4.4 ± 0.8	0 (0)	4.4 ± 0.9	2 (2.3)	0.775
HDL in mmol/L	1.3 ± 0.4	4 (1.1)	1.2 ± 0.3	1 (0.5)	1.3 ± 0.4	2 (2.3)	0.071
Cholesterol/HDL ratio	3.6 ± 1.1	160 (44.3)	3.7 ± 1.4	57 (31.0)	3.4 ± 1.0	35 (39.8)	0.185
LDL in mmol/L	2.4 ± 0.9	12 (3.3)	2.4 ± 0.8	3 (1.6)	2.3 ± 0.8	3 (3.4)	0.473
Triglycerides in mmol/L	1.7 ± 1.0 1.5 (1.0–2.1)	7 (1.9)	1.8 ± 1.3 1.5 (1.1–2.1)	0 (0)	1.7 ± 1.0 1.4 (1.0–2.0)	2 (2.3)	0.531
Creatinine in *µ*mol/L	78.6 ± 17.2 77.0 (67.0–88.0)	6 (1.7)	80.9 ± 17.4 79.0 (68.0–92.0)	1 (0.5)	77.8 ± 17.8 75.5 (66.0–85.8)	0 (0)	0.259
Alb./creat. ratio in mg/mmol							
Men	2.0 ± 4.4 0.7 (0.3–1.5)	23 (10.7)	2.2 ± 6.9 0.5 (0.3–1.5)	18 (15.9)	1.3 ± 1.7 0.7 (0.3–1.5)	7 (14.0)	0.636
Women	1.6 ± 3.5 0.7 (0.3–1.5)	31 (21.1)	1.1 ± 1.3 0.7 (0.4–1.5)	8 (11.3)	0.6 ± 0.5 0.6 (0.3–0.9)	10 (26.4)	0.155
MDRD in mL/min/1.73 m^2^	79.1 ± 49.0 75.0 (61.0–88.0)	5 (1.4)	75.7 ± 16.2 73.0 (61.0–87.0)	1 (0.5)	76.7 ± 17.4 75.5 (61.0–89.0)	0 (0)	0.610
Smoking							
Yes	54 (15.1)	3 (0.8)	30 (16.4)	1 (0.5)	11 (12.5)	0 (0)	0.382
Before	158 (44.1)		73 (39.7)		31 (35.2)		
No	146 (40.8)		80 (43.7)		46 (52.3)		
Alcohol consumption in units/day							
0	166 (58.9)	79 (21.9)	98 (60.9)	23 (12.5)	41 (60.3)	20 (22.7)	0.646
1	61 (21.6)		8 (23.6)		14 (20.6)		
2	39 (13.8)		17 (10.6)		13 (19.1)		
3	11 (3.9)		7 (4.3)		0 (0)		
4	4 (1.4)		1 (0.5)		0 (0)		
5	0 (0)		0 (0)		0 (0)		
6	1 (0.4)		0 (0)		0 (0)		
Employment							
Fulltime/part-time working	99 (34.3)	72 (19.9)	61 (39.6)	30 (16.3)	20 (23.8)	4 (4.5)	0.063
Retired	134 (46.4)		70 (45.5)		46 (54.8)		
Unemployed/housekeeper	38 (13.1)		20 (13.0)		9 (10.7)		
Incapacitated	18 (6.2)		3 (1.9)		9 (10.7)		
Educational level							
None	0 (0)	73 (20.2)	1 (0.7)	31 (16.8)	0 (0)	4 (4.5)	0.125
Primary school	24 (8.3)		9 (5.9)		4 (4.8)		
Low	127 (44.1)		52 (34.0)		27 (32.1)		
Intermediate	86 (29.9)		51 (33.3)		30 (35.7)		
High	51 (17.7)		40 (26.1)		23 (27.4)		

**Table 10:** Medication prescription of curious users, active users, and nonusers.

Medication prescription *n* (%)	Nonusers (*n* = 361)	Missing	Curious users (*n* = 184)	Missing	Active users (*n* = 88)	Missing	Univariate *p* value
Diabetes-related							
Oral treatment only	251 (71.3)	9 (2.5)	128 (71.5)	5 (2.7)	64 (72.7)	0 (0)	1.000
Insulin treatment only	4 (1.1)	9 (2.5)	1 (0.5)	5 (2.7)	0 (0)	0 (0)	0.899
Mix of oral and insulin treatment	40 (11.4)	9 (2.5)	18 (10.1)	5 (2.7)	5 (5.7)	0 (0)	0.242
No medication	57 (16.2)	9 (2.5)	32 (17.9)	5 (2.7)	19 (21.6)	0 (0)	0.521
Comorbidity or complication related							
Calcium channel blockers	50 (14.2)	9 (2.5)	31 (17.3)	5 (2.7)	16 (18.2)	0 (0)	0.415
Beta blockers	128 (36.4)	9 (2.5)	74 (41.3)	5 (2.7)	36 (40.9)	0 (0)	0.324
Diuretics	121 (34.4)	9 (2.5)	63 (35.2)	5 (2.7)	31 (35.2)	0 (0)	0.979
Ace and RAAS inhibitors	196 (55.7)	9 (2.5)	94 (52.5)	5 (2.7)	47 (53.4)	0 (0)	0.738
Other blood pressure lowering medications	3 (0.9)	9 (2.5)	0 (0)	5 (2.7)	1 (1.1)	0 (0)	0.357
Lipid lowering medication	280 (79.5)	9 (2.5)	141 (78.8)	5 (2.7)	72 (81.8)	0 (0)	0.868

**Table 11:** Complications and risk factors of curious users, active users, and nonusers.

Complications and risk factors *n* (%)	Nonusers (*n* = 361)	Missing	Curious users (*n* = 184)	Missing	Active users (*n* = 88)	Missing	Univariate *p* value
Cardiovascular, total	225 (96.2)	127 (35.2)	128 (98.5)	54 (29.3)	59 (98.3)	28 (31.8)	0.506
Cardiovascular, specific							
Angina pectoris	41 (21.7)	172 (47.6)	21 (21.4)	86 (46.7)	7 (15.9)	44 (50.0)	0.698
Myocardial infarct	29 (15.3)	172 (47.6)	15 (15.3)	86 (46.7)	8 (17.4)	42 (47.7)	0.932
Other/chronic ischemic heart diseases	34 (16.1)	150 (41.6)	18 (14.9)	63 (34.2)	6 (10.9)	33 (37.5)	0.750
Hypertension	191 (84.1)	134 (37.1)	113 (93.4)	63 (34.2)	51 (92.7)	33 (37.5)	**0.025**
TIA	12 (6.4)	174 (48.2)	4 (4.2)	88 (47.8)	3 (7.0)	45 (51.1)	0.747
CVA	13 (7.0)	176 (48.8)	6 (6.1)	86 (46.7)	4 (9.5)	46 (52.3)	0.745
Intermittent claudication	7 (3.3)	150 (41.6)	4 (3.3)	63 (34.2)	3 (5.5)	33 (37.5)	0.689
Aortic aneurysms	4 (1.9)	150 (41.6)	1 (0.8)	63 (34.2)	1 (1.8)	33 (37.5)	0.731
CABG	15 (5.1)	68 (18.8)	7 (5.0)	43 (23.4)	4 (6.3)	25 (28.4)	0.916
PTCA	28 (9.6)	68 (18.8)	9 (6.4)	43 (23.4)	5 (7.8)	24 (27.3)	0.588
Heart failure	14 (8.1)	189 (52.4)	9 (9.4)	88 (47.8)	1 (2.5)	48 (54.5)	0.409
Retinopathy	19 (9.3)	156 (43.2)	14 (11.7)	64 (34.8)	4 (7.0)	31 (35.2)	0.640
Renal impairment	35 (18.6)	173 (47.9)	15 (15.5)	87 (47.3)	11 (25.6)	45 (51.1)	0.350
Albuminuria							
Men	30 (14.5)	7 (3.3)	14 (12.6)	2 (1.8)	6 (12.5)	2 (4.0)	0.908
Women	8 (5.8)	8 (5.4)	1 (1.4)	1 (1.4)	0 (0)	4 (10.5)	0.226
Neuropathy	49 (22.2)	140 (38.8)	30 (24.6)	62 (33.7)	9 (17.3)	36 (40.9)	0.594
Foot complication							
SIMMs 0	228 (77.6)	67 (18.6)	105 (73.4)	41 (22.3)	56 (83.6)	21 (23.9)	0.524
SIMMs 1	57 (19.4)		31 (21.7)		9 (13.4)		
SIMMs 2 or 3	9 (3.1)		7 (4.9)		2 (3.0)		
Psychiatric disorders	19 (9.0)	150 (41.6)	7 (5.8)	63 (34.2)	2 (3.6)	33 (37.5)	0.317

**Table 12:** Scores on quality of care (Europep) of curious users, active users, and nonusers.

Items of Europep: patients who scored 4 (good) to 5 (excellent) *n* (%)	Nonusers (*n* = 361)	Missing	Curious users (*n* = 184)	Missing	Active users (*n* = 88)	Missing	Univariate *p* value
*What is your assessment of the general practitioner over the last 12 months with respect to the following? *							
Making you feel that you have time during consultation	737 (95.1)	105 (11.9)	174 (99.4)	9 (4.9)	82 (95.3)	2 (2.3)	0.159
Showing interest in your personal situation	707 (91.6)	108 (12.3)	165 (94.8)	10 (5.4)	81 (94.2)	2 (2.3)	0.970
Making it easy for you to tell him or her about your problem	694 (92.2)	127 (14.4)	165 (98.2)	16 (8.7)	80 (93.0)	2 (2.3)	**0.047**
Involving you in decisions about your medical care	680 (90.7)	130 (14.8)	159 (93.5)	14 (7.6)	80 (95.2)	4 (4.5)	0.489
Listening to you	709 (92.0)	109 (12.4)	163 (94.8)	12 (6.5)	80 (94.1)	3 (3.4)	0.579
Keeping your records and data confidential	661 (93.8)	175 (19.9)	161 (97.0)	18 (9.8)	75 (93.8)	8 (9.1)	0.764
Providing quick relief of your symptoms	588 (83.9)	179 (20.3)	137 (86.2)	25 (13.6)	64 (85.3)	13 (14.8)	0.863
Helping you to feel well so that you can perform your normal daily activities	599 (89.7)	212 (24.1)	135 (91.8)	37 (20.1)	61 (89.7)	20 (22.7)	0.780
Thoroughness of the approach to your problems	675 (90.7)	136 (15.5)	153 (90.5)	15 (8.2)	74 (88.1)	4 (4.5)	0.769
Your physical examination	639 (90.8)	176 (20.0)	153 (92.7)	19 (10.3)	69 (90.8)	12 (13.6)	0.577
Offering services for preventing diseases (screening, health checks, and immunizations)	653 (92.5)	174 (19.8)	151 (91.5)	19 (10.3)	74 (94.9)	10 (11.4)	0.539
Explaining the purpose of examinations, tests, and treatments	677 (92.4)	147 (16.7)	163 (94.8)	12 (6.5)	77 (91.7)	4 (4.5)	0.402
Telling you enough about your symptoms and/or illness	662 (90.2)	146 (16.6)	158 (92.4)	13 (7.1)	80 (95.2)	4 (4.5)	0.579
Helping you deal with emotions related to your health status	424 (84.8)	380 (43.2)	89 (86.4)	81 (44.0)	44 (81.5)	34 (38.6)	0.659
Helping understand why it is important to follow the GP's advice	631 (89.1)	172 (19.5)	146 (89.6)	21 (11.4)	73 (89.0)	6 (6.8)	0.965
Knowing what has been done or told during previous contacts in the practice	601 (86.8)	188 (21.4)	148 (90.2)	20 (10.9)	71 (87.7)	7 (8.0)	0.158
Preparing you for what to expect from specialists, hospital care, and other care providers	424 (82.8)	368 (41.8)	107 (8.3)	54 (29.3)	49 (86.0)	31 (35.2)	0.577

*What is your assessment of the general practice over the last 12 months with respect to the following? *							
The helpfulness of the practice staff (other than the doctor) to you	313 (93.4)	26 (7.2)	158 (92.9)	14 (7.6)	77 (92.8)	5 (5.7)	0.953
Getting an appointment to suit you	301 (88.5)	21 (5.8)	152 (86.9)	9 (4.9)	72 (85.7)	4 (4.5)	0.867
Getting through to the practice on telephone	249 (73.0)	20 (5.5)	120 (68.6)	9 (4.9)	60 (71.4)	4 (4.5)	0.777
Being able to talk to the general practitioner on the telephone	167 (70.5)	124 (34.3)	71 (62.8)	71 (38.6)	35 (63.6)	33 (37.5)	0.306
Waiting time in the waiting room	246 (71.3)	16 (4.4)	119 (68.4)	10 (5.4)	51 (59.3)	2 (2.3)	0.148
Providing quick services for urgent health problems	241 (90.3)	94 (26.0)	118 (86.8)	48 (26.1)	53 (86.9)	27 (30.7)	0.658

**Table 13:** Scores on quality of life (EQ-5D), well-being (WHO-5), diabetes-related distress (PAID-5), and self-care behavior (SDSCA) of curious users, active users, and nonusers.

EQ-5D, WHO-5, PAID-5, and SDSCA *n* (%)/mean ± SD/median (25–75 quartiles)	Nonusers (*n* = 361)	Missing	Curious users (*n* = 184)	Missing	Active users (*n* = 88)	Missing	Univariate *p* value
EQ-5D index-score	0.9 ± 0.2	72 (19.9)	0.9 ± 0.1	32 (17.4)	0.9 ± 0.2	4 (4.5)	**0.030**
EQ-VAS	74.7 ± 17.4 80.0 (60.0–90.0)	74 (20.5)	79.3 ± 13.8 80.0 (73.0–90.0)	35 (19.0)	76.9 ± 16.5 80.0 (0.0–90.0)	5 (5.7)	**0.032**
WHO-5 index-score	70.4 ± 17.9 76.0 (60.0–80.0)	76 (21.1)	74.1 ± 12.7 76.0 (68.0–80.0)	33 (17.9)	70.2 ± 16.5 76.0 (60.0–80.0)	1 (1.1)	0.080
WHO-5 score indicates suboptimal well-being, screening depression advised	36 (12.6)	76 (21.1)	8 (5.3)	33 (17.9)	9 (10.8)	1 (1.1)	**0.018**
WHO-5 answers advise screening depression	43 (15.5)	76 (21.1)	6 (4.0)	33 (17.9)	11 (13.3)	1 (1.1)	**0.002**
PAID-5 total score	2.8 ± 3.1 2.0 (0.0–4.5)	76 (21.1)	1.8 ± 2.4 1.0 (0.0–3.0)	32 (17.4)	2.2 ± 2.5 1.0 (0.0–4.0)	1 (1.1)	**0.016**
PAID-5 score indicates distress	15 (5.3)	76 (21.1)	4 (2.6)	32 (17.4)	2 (2.4)	1 (1.1)	0.183
SDSCA							
General diet in number of days	5.4 ± 1.8 6.0 (5.0–7.0)	76 (21.1)	5.5 ± 1.9 6.0 (5.0–7.0)	32 (17.4)	5.8 ± 1.7 6.0 (5.5–7.0)	5 (5.7)	0.258
Specific diet in number of days	5.6 ± 1.1 5.7 (4.7–6.3)	73 (20.2)	5.7 ± 1.0 6.0 (5.0–6.7)	30 (16.3)	5.7 ± 1.0 6.0 (5.3–6.6)	4 (4.5)	0.160
Exercise in number of days	4.0 ± 2.0 4.0 (2.5–5.5)	72 (19.9)	4.1 ± 1.8 4.0 (2.9–5.6)	30 (16.3)	3.8 ± 1.8 3.8 (2.5–5.0)	4 (4.5)	0.612
Blood-glucose in number of days	2.1 ± 2.2 1.0 (0.0–4.0)	74 (20.5)	2.1 ± 2.4 1.0 (0.0–4.0)	30 (16.3)	1.8 ± 1.8 1.0 (0.5–2.3)	4 (4.5)	0.241
Foot-care in number of days	1.9 ± 2.0 1.5 (0.0–3.5)	72 (19.9)	1.9 ± 2.0 1.0 (0.0–3.5)	30 (16.3)	1.8 ± 2.0 1.0 (0.0–3.5)	4 (4.5)	0.924
Medication in number of days	6.7 ± 1.0 7.0 (7.0–7.0)	73 (20.2)	7.0 ± 0.2 7.0 (7.0–7.0)	30 (16.3)	6.8 ± 0.8 7.0 (7.0–7.0)	4 (4.5)	**0.020**
Smoking	54 (25.1)	146 (40.4)	24 (21.8)	74 (40.2)	14 (24.6)	31 (35.2)	0.704

## Appendices

### A. Results of Users and Nonusers of the Online Care Platform e-Vita

See Tables 4, 5, 6, 7, and 8.

### B. Results of Curious Users, Active Users, and Nonusers of the Online Care Platform e-Vita

See Tables 9, 10, 11, 12, and 13.

## Abbreviations

EQ-5D: EuroQol-5 Dimensions
EQ-VAS: EuroQol Visual Analogue Scale
GFR: Glomerular filtration rate
GP: General practitioner
HRQoL: Health-related quality of life
MDRD: Modification of Diet in Renal Disease
PAID-5: Problem Areas in Diabetes-5 questions
PN: Practice nurse
SDSCA: Summary of Diabetes Self-Care Activities
T2DM: Type 2 diabetes mellitus
WHO-5: WHO-Five Item Measure of Well-Being.
